# Do childhood cognitive ability or smoking behaviour explain the influence of lifetime socio-economic conditions on premature adult mortality in a British post war birth cohort?^☆^

**DOI:** 10.1016/j.socscimed.2009.02.006

**Published:** 2009-05

**Authors:** Diana Kuh, Imran Shah, Marcus Richards, Gita Mishra, Michael Wadsworth, Rebecca Hardy

**Affiliations:** MRC Unit for Lifelong Health and Ageing, Department of Epidemiology and Public Health, Royal Free and University College Medical School, 33 Bedford Place, London WC1B 5JU, United Kingdom

**Keywords:** Mortality, Socio-economic conditions, Childhood cognitive ability, Cigarette smoking, Birth cohort study, Life course, UK

## Abstract

Poor childhood and adult socio-economic conditions, lower childhood cognitive ability and cigarette smoking are all associated with adult mortality risk. Using data on 4458 men and women aged 60 years from a British birth cohort study, we investigated the extent to which these risk factors are part of the same pathway linking childhood experience to adult survival. Compared with women from non-manual origins, men from non-manual origins, women and men from manual origins, and those with missing data on father's social class had about double the risk of mortality between 26 and 60 years. Cox proportional hazards models showed that these differences were reduced but remained significant after adjusting for childhood cognitive ability, adult socio-economic conditions and smoking. Higher childhood ability increased survival chances by securing better adult socio-economic conditions, such as home ownership, which was strongly associated with survival. These findings were similar for cardiovascular and cancer mortality.

## Introduction

Childhood socio-economic conditions are associated with adult mortality risk in historical and contemporary populations (Bengtsson & Brostrom, 2006; Elo & Preston, 1992; Lawlor, Sterne, Tynelius, Davey Smith, & Rasmussen, 2006; Osler et al., 2003). In the British 1946 birth cohort we found an almost five-fold difference in survival between ages 26–54 years for those in the most advantageous social conditions in childhood and early adulthood, compared with those in the least advantageous conditions during this time (Kuh, Hardy, Langenberg, Richards, & Wadsworth, 2002). Other studies suggest that these effects are particularly consistent in relation to cardiovascular (Galobardes, Lynch, & Davey Smith, 2004; Pollitt, Rose, & Kaufman, 2005) and respiratory (Davey Smith, Hart, Blane, Gillis, & Hawthorne, 1997) mortality.

Within a life course framework, several compatible pathways have been identified as central to the link between child disadvantage and poor adult health (Power & Kuh, 2006); poor physical and mental health earlier in life, slower cognitive development and educational progress, health damaging behaviours, and lifetime continuity in social disadvantage (Power & Kuh, 2006). Of these, education may be particularly important, as a mediator of the effects of early disadvantage (Blane, 2003), and as a path of access to resources that influence exposure to, or impact of, risks to morbidity and mortality (Mirowsky & Ross, 2003; Pearlin, 1999; Phelan & Link, 2005). However, cognition has been claimed to account for at least some of these processes, because it reflects integrity of biological processes underlying health (Whalley & Deary, 2001), and because it is part of a causal chain linking childhood socio-economic conditions to educational and socio-economic attainment (Neisser et al., 1996; Richards & Sacker, 2003). In this regard, studies have shown associations between childhood or adult cognition and all cause and cause specific mortality (Deary & Der, 2005; Hemmingsson, Melin, Allebeck, & Lundberg, 2006; Osler et al., 2003; Shipley, Der, Taylor, & Deary, 2006; Whalley & Deary, 2001). The evidence to date does not suggest that childhood cognition mediates the effects on mortality of early socio-economic conditions (Deary & Batty, 2006). Whether the effects of childhood cognition and adult socio-economic conditions are independently related to mortality, or are part of the same causal pathway is not yet elucidated. In the British 1946 birth cohort, the effect of childhood cognitive ability on mortality risk in men to age 54 was largely explained by socio-economic disadvantage in early adult life (Kuh, Richards, Hardy, Butterworth, & Wadsworth, 2004).

Smoking is potentially an important causal link on the pathway between lifetime socio-economic circumstances, cognition and premature mortality. It is the biggest single cause of premature death in the industrialised world (Peto, Lopez, Boreham, Thun, & Heath, 1992), and shows socio-economic (Townsend, Roderick, & Cooper, 1994) and cognitive (Taylor et al., 2003) gradients, although has failed so far to explain the early socio-economic conditions–mortality association (Davey Smith, Hart, Blane, & Hole, 1998; Davey Smith, McCarron, Okasha, & McEwen, 2001; Gliksman et al., 1995; Kuh et al., 2002) or the cognition-mortality association (Deary & Der, 2005; Kuh et al., 2004). Using the 1946 birth cohort we update and extend our previous analyses on the British 1946 birth cohort by investigating the effects of lifetime socio-economic conditions, childhood cognition, and smoking on mortality between 26 and 60 years, and the extent to which they are part of the same pathway linking childhood experience to adult survival. For the first time we provide information on cardiovascular and cancer mortality in relation to these risk factors.

## Methods

The Medical Research Council's National Survey of Health and Development is a prospective national cohort of 2547 women and 2815 men, a socially stratified sample of all births that took place in England, Scotland and Wales during the week 3–9 March 1946 (Wadsworth et al., 2003). Start of follow-up was taken as 1971, when cohort members were age 26 years and were flagged for death on the National Health Service Central Register. By then 4458 men and women were alive and resident in Britain and available for analysis; of the remaining cohort, 881 had already died or emigrated and a further 27 were excluded because they were not flagged on the Central Register.

The underlying cause of death between 26 and 60 years was coded using either ICD9 or ICD10 disease classifications according to standard rules, which distinguished mortality from cardiovascular diseases (ICD9 codes 401-454 and ICD10 codes I10-I89) and cancers (ICD9 codes 140-239 and ICD10 codes C00-C97).

Indicators of childhood socio-economic conditions, based on information provided by the mother at home interviews undertaken by health visitors, included father's social class at age 4 years (manual/non-manual) and parental education (more than a primary education or not). A score of housing quality (range 0–3) allocated one point for each of the following: dwelling in very good repair, dwelling built since 1919, no overcrowding (no more than one and a half persons per room). Those who scored 0 and those scoring 1 were compared with those scoring 2 or 3 (the best conditions). A score of care of the home and child (range 0–5) allocated one point for each of the following ratings by the health visitor: very clean house, very clean child, at least adequate shoes, at least adequate clothes, mother coped well. Those who scored 0–2 and those scoring 3 or 4 were compared with those scoring the highest (the best care).

Indicators of adult socio-economic conditions, based on information provided by the cohort member at ages 26, 36, 43 and 53 years, were two measures of household social class (one based on the man's occupation and a gender neutral measure based on the occupation given the highest social class), net income (bottom third/top two thirds) and home ownership (yes/no).

Childhood cognitive ability was measured at age 8 years, 11 and 15 years using tests designed by the National Foundation of Education Research. These tests have been described in detail elsewhere (Richards, Shipley, Fuhrer, & Wadsworth, 2004). At age 8 children were tested on reading comprehension, word pronunciation, vocabulary, and non-verbal reasoning; at age 11 on verbal and non-verbal intelligence, arithmetic, word pronunciation, and vocabulary; at age 15 on verbal and non-verbal intelligence (the AH4 test), reading comprehension, and mathematics (Pigeon, 1964; Pigeon, 1968). All scores for each test were standardised (with a mean score of 0 and a standard deviation of 1) and divided into four equal groups with the lowest quarter being the reference group. Total scores representing overall cognitive function at ages 8,11, and 15 were obtained by standardising the sum of these scores. Avoidable losses discussed elsewhere (Douglas, 1964) were due to study members being untraced, absent from school, not mentally able to take the tests, having parents who refused to let them take the tests, or attending schools which did not set aside time for testing.

Educational progress was measured by the level of qualifications obtained at age 26 years. Men and women with degrees and other higher level qualifications were grouped with those who left school (usually at age 18 years) with advanced secondary qualifications as there were no deaths in women with the highest level of qualifications. Men and women who gained ordinary level qualifications (usually taken at age 16 years) were distinguished from those who left school with no qualifications.

Smoking status was defined by the most recent behaviour identified for each individual before death or censoring occurred and was categorised as never smoker, former smoker or current smoker. Never smokers were taken as the reference group.

Table 1 gives the socio-economic characteristics for the whole sample. Fifty five percent of the survey members were from a manual background in childhood, while 38% were in a manual occupation during adulthood.

### Analysis

Survival curves, obtained using the Kaplan–Meier method, were first used to compare the cumulative death rate between 26 and 60 years for those in the most socio-economically disadvantaged groups in childhood and adulthood with the rate for the most advantaged group. Cox's proportional hazards models were used to investigate the relationships between socio-economic conditions and adult mortality rates in the whole sample, and in men and women separately. Those with missing data were included as a separate group but were excluded in the analyses stratified by sex because of the small number of deaths in these groups. In all analyses, the highest socio-economic group was taken as reference. The proportional hazards assumption was checked. Follow-up time (in months) was from the cohort's 26th birthday until the first of death, emigration, or the end of March 2006, the cohort's 60th birthday. If death had not occurred, follow-up was treated as censored. For adult socio-economic indicators, the most recent measure recorded for each individual before death or censoring occurred was used in analyses.

Using all cause mortality as the outcome, we included father's social class and the most powerful adult socio-economic measures in the same model, identified by including various pairs of adult indicators in hazards models and seeing which had the strongest independent effects on mortality. We tested whether the patterns seen for all cause mortality were also seen for cardiovascular mortality and cancer mortality using competing risks Cox's proportional hazards models, which involved censoring deaths from other causes, at time of death. We tested for sex interactions and, where significant, report the results for men and women separately in the text.

We then investigated whether childhood cognitive ability and educational qualifications were related to all cause mortality, and if these relationships were independent after mutual adjustment. We tested whether they accounted for the effects of childhood or adult conditions on all cause mortality. Finally, we investigated the effect of smoking on all cause mortality and tested whether smoking accounted for the effects of socio-economic conditions or childhood cognitive ability on mortality. These analyses were repeated using cardiovascular disease mortality and cancer mortality as outcomes.

All models were conducted in STATA 8.2 and repeated allowing for the initial sampling procedure. As the results of these models were very similar, the unweighted results are presented.

## Results

Between age 26 and 60 years 332 people (137 women and 195 men) died. The death rate was higher for men compared with women (hazard ratio (HR) 1.3, 95% confidence intervals 1.1, 1.7).

### Socio-economic conditions in childhood and adulthood

**Fig. 1:**
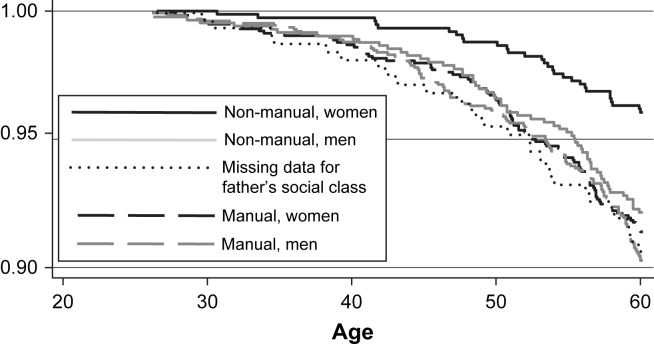
Survivor functions by father's social class and gender based on 4461 study members and 332 deaths.

By 60 years, study members from manual origins were 60% more likely to have died (8.7%) than those from non-manual origins (5.4%) (log rank test = .005). This was confirmed by sex-adjusted estimates from a Cox's proportional hazards model (Table 2). There was an effect of father's social class on adult mortality for women (HR = 2.2, 95% CI:1.5,3.4) but not for men (HR = 1.2, 95% CI:0.9,1.7), and this difference was significant (*p*-value = .03 for the interaction between sex and father's social class). Men from non-manual origins, women and men from manual origins, and those with missing data on father's social class had about double the risk of adult mortality compared with women from non-manual origins (Fig. 1). A variable distinguishing these five groups, with non-manual women as the reference group as they have the lowest mortality rate, was used in further analyses. Those with missing data on father's social class included those where the father was absent, had died, or where the mother was not interviewed at age 4 years. These study members had death rates similar to those from the manual class.

The hazard ratios (worst versus best) for the scores for housing quality and care of house and child were similar to the ratio associated with social class of origin (Table 2). The effects of paternal or maternal education were smaller. When each of these indicators was added separately to a model containing father's social class including missing categories and gender, the hazard ratios (worst versus best) for housing quality and for care of the house and child remained associated with mortality. In these models, father's social class remained associated with mortality (not shown).

Study members living in manual households in adult life had a 60% greater death rate of those in non-manual households (HR = 1.6, 95% CI:1.3,2.1) (Table 2). The gender neutral measure of adult social class gave similar results. Those not owning their own home at 26 years had almost a tripling of the death rate compared to those who did (HR = 2.8, 95% CI:2.2,3.5). Those in the bottom third of household income had an 80% higher death rate compared with those in the top two thirds. The effect of household income was accounted for by home ownership. The effect of being in a manual household was also attenuated after accounting for home ownership but social class continued to be included in further models as those missing a social class had a raised mortality risk (not shown).

In a model containing the groups based on father's social class and gender, and adult household social class and home ownership, women from non-manual origins retained their significantly lower mortality rates over others even after accounting for both indicators of adult socio-economic conditions (Table 3). In this model, there were no additional effects of childhood housing quality or care of the house and child

Of the 332 deaths between 26 and 60 years, 81 (24.4%) were from cardiovascular diseases (CVD) and 136 (41.0%) were from cancers. The effects of socio-economic conditions were in the same direction for CVD and cancer mortality (Table 3). The socio-economic effects appeared strongest for CVD mortality but the cause specific models have low power due to the smaller number of deaths and their relative strengths are difficult to assess. For CVD mortality the effect of father's social class was stronger for women than for men, as for all cause mortality (in sex-specific models, HR = 3.7, 95% CI:1.3,10.8 for women from manual compared with non-manual origins, and HR = 1.3, 95% CI:0.71,2.4, for men from manual compared with non-manual origins; *p*-value for interaction = .09). For cancer mortality, there was an effect of father's social class for men (HR = 2.4, 95% CI:1.3,4.4, *p* = .005) as well as women (HR = 2.0, 95% CI:1.1,3.6, *p* = .02).

### Childhood cognitive ability and educational qualifications

Higher scores on the cognitive tests at 8, 11 and 15 years were associated with lower adult mortality (Table 4). Those with missing data at any age had death rates that were similar to those in the lowest quarter of the score. These standardised scores at 8, 11 and 15 years were strongly correlated (Pearson correlations between 0.76 and 0.88), and so the scores at age 15 years for those with missing data at that age were imputed from their standardised scores at earlier ages, if available, and used in further analyses (Table 4). By 60 years, those in the lowest quarter of this score were almost twice as likely to have died (10.4%) than those in the top quarter (5.4%)(log rank test = .0004).

Increasing levels of educational qualifications by age 26 were also associated with lower adult mortality (Table 4). In a model including both cognitive ability and educational qualifications, neither indicator predominated and each confounded the other (not shown).

The cognitive score was strongly related to father's social class, household social class and home ownership (all *p* < .001). When entered into a model with father's social class and gender (model b, Table 5), the adverse effects of a lower cognitive score and of coming from manual origins were attenuated although both remained associated with higher mortality. Including adult social class and home ownership (model c, Table 5) slightly weakened the effect of father's social class and gender; the effect of the cognitive score was strongly attenuated and no longer significant. Similar results were obtained when cognitive ability was fitted as a continuous score and when educational qualifications were substituted for cognitive ability.

The results of the multivariable analyses for cancer and CVD mortality were similar to all cause mortality in that the effects of cognition were strongly attenuated after adjusting for childhood and adult socio-economic indicators (not shown).

### Smoking behaviour

By 60 years, study members who were smokers at last contact were over twice as likely to have died (11.7%) than non-smokers (5.1%) (log rank test = <.001). Smokers had a HR of 2.4 (95% CI:1.8,3.1) compared with never smokers. The effects were stronger in women (HR = 3.2, 95% CI:2.1,4.8) than in men (HR = 1.9, 95% CI:1.3,2.7, *p* = .17 for the interaction between smoking and sex). When smoking was added to the separate multivariable models for men and women (Tables 6a and b), these estimates and the estimates for the effects of adult socio-economic indicators on mortality were slightly reduced. For women, smoking and not owning a home were the strongest predictors of mortality but father's social class was also independently associated with mortality. For men, not owning a home, and to a slightly lesser extent smoking, were the strongest predictors of mortality.

Smoking was strongly associated with cardiovascular mortality (for women, HR = 3.1, 95% CI:1.2,7.8, *p* = .016, for men, HR = 3.1, 95% CI:1.5,6.6, *p* = .003) and cancer mortality (for women, HR = 3.1, 95% CI:1.8,5.6, *p* < .001, for men, HR = 2.5, 95% CI:1.3,4.6, *p* = .005) and its effects in the multivariable models were similar to its effects for all cause mortality (not shown).

## Discussion

In a British cohort born immediately after the second world war, the effects of childhood socio-economic conditions on adult premature mortality were strong in women but not in men. This sex-specific pattern had been evident, but not as strong, by 54 years (Kuh et al., 2002). In contrast, being in the manual social class, not owning your own home and lower household income in adulthood had similarly strong adverse effects on mortality in men and women. The effect of cognitive ability at age 8 on mortality had been seen by age 54 years, but increased mortality rates were only observed in men in the lowest quarter of ability. By age 60 years, and using the results of tests taken at age 15, the effect was evident for women as well as men and operated across the full range of ability. After adjustment for adult socio-economic conditions, particularly home ownership, the effect of cognitive ability on mortality was strongly attenuated, but the effect of childhood socio-economic conditions on mortality in women remained. In turn, the effect of adult socio-economic conditions on mortality was partly accounted for by smoking behaviour. This pattern of predictors shown for all cause mortality was also seen for cardiovascular and cancer mortality.

### Limitations and advantages of our study

The main limitation of our study is its lack of statistical power, particularly for cause specific mortality analyses, due to the small numbers of deaths. Many of the variables were dichotomised a priori to analyses in order to ensure that there were adequate number of deaths in each category for a meaningful comparison. Smoking status was limited by the reliance on self-reports. The study's main advantages are the range of socio-economic indicators from childhood and adult life, the availability of measures of cognition and education, its inclusion of women as well as men in a nationally representative sample, and the mortality follow-up of those with missing explanatory variable data.

### Lifetime socio-economic conditions and premature mortality

The stronger effect of early socio-economic conditions on premature mortality for women but not men is a key finding in this cohort study. A large record linkage study of Swedish men and women also reported a sex differential in the effect of childhood social class on all cause mortality, but found a significantly larger effect for men than for women (for men, HR = 1.31, 95% CI:1.29,1.34, for women, HR = 1.18, 95% CI:1.15,1.21) (Lawlor et al., 2006). This differential effect was driven by a stronger effect of early social class on mortality from injury and poisoning, mental disorders and alcoholic cirrhosis in men. We have less power to detect social differentials in these causes of death because our sample size is much smaller and there were few deaths from these causes, and because of a later age at start of mortality follow-up (26 years compared to 10 years). This may have reduced the effect of early social class on premature mortality in men in our study. However, this does not explain why the effect of early social class on mortality in Swedish women was so much lower than the effect we found for British women. One possible reason is that inequality in opportunity (as assessed by early social class) for British women born in 1946 has been much greater than inequality in opportunity for Swedish women born between 1944 and 1960, and this has translated into a greater differential in adult mortality. As our study suggests that this differential is only partly explained by childhood cognition, adult socio-economic conditions and smoking, other explanations must be sought elsewhere. Other studies have implicated additional behavioural and emotional processes (Hemmingsson & Lundberg, 2005) as explanations for childhood socio-economic differences in mortality and we plan to explore these, and see whether they account for the greater vulnerability of women to early disadvantage.

The persistent protective effect of home ownership on mortality, even after adjustment for cognitive ability and smoking, may be because it more accurately reflected socio-economic conditions throughout life than a one off measure of social class. Home ownership reflects past, current and future wealth, and home owners in this cohort benefited from the rapidly rising British house prices since the 1970s (Halsey & Webb, 2000). This may explain why its effect on mortality was less reduced by adjusting for adolescent cognitive and behavioural characteristics of the study member than the effects of other early adult socio-economic indicators. Another possibility is that housing tenure reflects access to socio-economic resources that operate at the contextual level, since property ownership may be associated with health-enhancing neighbourhood advantages, such as control of pollution, that do not exclusively depend on individual initiative (Phelan & Link, 2005). By age 53 years, home ownership was common (87%) and lack of home ownership probably identified a particularly disadvantaged group. To investigate whether duration of ownership mattered we undertook additional analyses (not reported) that tested whether mortality rates varied for home owners depending on whether they had first reported home ownership at 26, 36, 43 or 53 years. We found no evidence that early home ownership was particularly advantageous but these analyses were underpowered because of the small number of deaths in some groups. However, these findings, and our previous report based on home ownership at 26 years (Kuh et al., 2002) do suggest that the association between adult socio-economic conditions and mortality is not due to prior ill-health resulting in poorer adult socio-economic conditions.

Our findings show the importance of not relying only on measures of adult social class when elucidating pathways between lifetime socio-economic conditions and survival. The highest risk of mortality is not necessarily captured by a measure of social class; those for whom these data were missing, either because there was no occupation or because the occupation was unknown, had a particularly high risk of subsequent mortality in this study.

### Lifetime socio-economic conditions, cognition and education and premature mortality

The effect of early cognitive ability on adult mortality has been shown elsewhere (Batty, Mortensen, Nybo Andersen, & Osler, 2005; Hart et al., 2005; Hemmingsson et al., 2006; Martin & Kubzansky, 2005; O'Toole & Stankov, 1992; Osler et al., 2003; Whalley & Deary, 2001). Only a few of these studies (Batty, Der, MacIntyre, & Deary, 2006; Osler et al., 2003) have tested whether cognitive ability was an explanation for socio-economic differences in mortality. A study of a Danish birth cohort born in 1953 showed that the effects of early ability, as measured by scores on IQ tests taken at age 12 years, explained about a quarter of the effect of childhood socio-economic conditions on all cause mortality (Osler et al., 2003). In our study, childhood socio-economic conditions had a stronger effect on female mortality than cognitive ability; the latter was not a strong mediator of these socio-economic differences.

A study of 1347 men and women in the West of Scotland (Batty et al., 2006) showed that IQ measured in middle age strongly attenuated the effects of adult socio-economic position on mortality after sixteen years of follow-up. However this study did not report how adult socio-economic conditions changed the effect of IQ on mortality. Our study, that found that the mortality effects were stronger for adult socio-economic conditions than childhood cognitive ability, does not support these findings. Thus it would seem that higher childhood cognitive ability helps to secure healthier and safer socio-economic environments in adult life and, in turn, these increase survival chances. In due course our study will be powered to test whether adult cognitive ability measured at age 43 can further elucidate these pathways.

In a study of all Swedish individuals born between 1944 and 1960 and followed until 1990, adjustment for educational attainment resulted in a more marked attenuation of the effect of childhood socio-economic conditions on later life mortality than later life social class (Lawlor et al., 2006). The authors suggested that behavioural risk factors may be important mediators. We found no evidence that education was a mediator of the effect of childhood socio-economic conditions on mortality in women, and was no longer associated with mortality in men or women after home ownership and adult social class were taken into account. It may be that educational attainment based on qualifications is not a good discriminator when a third of both men and women in this cohort had no qualifications.

### Lifetime socio-economic conditions, smoking and premature mortality

The effects of smoking on mortality are well known, although there are substantially more data on men (Doll, Peto, Boreham, & Sutherland, 2004) than on women (Vollset, Tverdal, & Gjessing, 2006). There was some evidence in our study that the effect of smoking on all cause mortality was stronger in women than in men; other studies have shown that smoking is more detrimental in its effects on lung function and myocardial infarction in women than in men (Chen, Horne, & Dosman, 1991; Pope, Ashley, & Ferrence, 1999; Stabile & Siegfried, 2003). Smoking was a partial explanation for adult socio-economic differences in mortality but less of an explanation for childhood socio-economic differences in women. We might have expected smoking to account for more of the childhood socio-economic differences as a study across a number of cohorts, including the 1946 cohort, showed that smoking behaviour in women (but not in men) was influenced by social origins as well as destination (Power, et al., 2005).

The two-fold risk of all cause mortality from smoking in this cohort up to age 60 years is slightly lower than the three-fold difference in mortality in middle age (35–69 years) reported by Doll et al. (Doll et al., 2004) for smokers compared with non-smokers among male doctors born in the 1920s. Although our analysis was based on only one (the most recent) report of smoking behaviour, this was a reasonable indicator of lifelong smoking as the decrease in the proportion of smokers in the study between 26 and 59 years, matching the decrease in the wider population between 1972 and 2005, meant that those identified at the most recent contact were generally lifelong smokers.

Patterns of smoking by social class and gender vary over time within a population; variations across populations reflect the timing of the secular increase and subsequent decrease in smoking behaviours. Changing patterns in smoking behaviour are reflected in changing patterns of cause specific mortality (Strachan & Perry, 1997), and would explain why the strength of smoking as an explanation for social class mortality differences may differ in studies undertaken at different times or in different populations. In men, Doll et al. found that mortality risk for smokers born in the 1920s was greater than the risk for smokers born in the nineteenth century and reflected the earlier and more intensive use of cigarettes in professional men born in the 1920s. Women, and lower social class men took up the smoking habit somewhat later. In the 1946 birth cohort, smoking was common among men and women of all social classes when these cohort members were young adults in the 1960s. A higher proportion of those from non-manual households in childhood and adulthood, and a higher proportion of men than women have quit smoking in this cohort (Power et al., 2005), and smoking has increasingly become associated with socio-economic disadvantage. However, our findings suggest that smoking is not the primary cause of socio-economic mortality differences in this cohort.

## Conclusions

In an early post war cohort early socio-economic differences in all cause mortality were striking in women but not in men and were not fully explained by childhood cognitive ability, adult socio-economic conditions or smoking. The strongest adult socio-economic indicator of mortality in this cohort was home ownership. Higher childhood cognitive ability influenced mortality risk by helping to secure better adult socio-economic conditions, such as home ownership, which strongly improved survival chances. Better conditions were associated with a lower risk of smoking and this also improved survival, and partly explained socio-economic differences in mortality. These findings were similar for cardiovascular and cancer mortality.

## Figures and Tables

**Table 1:** Characteristics based on indicators of childhood (age 4 years), most recent adulthood socio-economic position, and smoking behaviour for the 4461 men and women.

	N (%)
**Socio-economic conditions at age 4 years**	
Father's social class	
Non-manual	1656 (37.1)
Manual	2457 (55.1)
*Missing*	*348* (*7.8*)

Mother's education	
Secondary level	890 (20.0)
Primary level only	3106 (69.6)
*Missing*	*465* (*10.4*)

Father's education	
Secondary level	1124 (25.2)
Primary level only	2824 (63.3)
*Missing*	*513* (*11.5*)

Housing quality	
Best	2163 (48.5)
Intermediate	1082 (24.3)
Worst	880 (19.7)
*Missing*	*336* (*7.5*)

Care of house and child	
Best	1569 (35.2)
Intermediate	1001 (22.4)
Worst	1274 (28.6)
*Missing*	*617* (*13.8*)

Educational qualifications by 26 y	
None	1993 (44.7)
Ordinary level	805 (18.1)
Advanced	1354 (27.1)
*Missing*	*309* (*6.9*)

**Most recent adult socio-economic conditions**	
Most recent household social class	
Non-manual	2346 (52.6)
Manual	1678 (37.6)
*Missing*	*437* (*9.8*)

Most recent housing tenure	
Owner occupier	3080 (69.0)
Other	901 (20.2)
*Missing*	*189* (*8.9*)

Most recent household income	
Top two thirds	2707 (60.7)
Bottom third	1183 (26.5)
*Missing*	*571* (*12.8*)

**Cigarette smoking**	
Never smoker	1602 (35.9)
Former smoker	1290 (28.9)
Current smoker	1292 (29.0)
*Missing*	*277* (*6.2*)

**Table 2:** Hazard ratios for mortality (26–60 years) for indicators of childhood (age 4 years) and most recent adulthood socio-economic position, obtained from Cox's proportional hazards models and based on 4461 men and women and 332 deaths.

	Total sample		Women	Men
	Hazard ratio (95% CI) sex adjusted	*p*-value^a^	Hazard ratio (95% CI) unadjusted	Hazard ratio (95% CI) unadjusted
**Socio-economic conditions at age 4 years**				
Father's social class				
Non-manual	1.0	<.001	1.0	1.0
Manual	1.6 (1.2,2.0)		2.2 (1.5,3.4)	1.2 (0.91,1.7)
*Missing*	*1.6* (*1.0,2.4*)			

Mother's education		.17		
Secondary level	1.0		1.0	1.0
Primary level only	1.2 (0.91,1.6)		1.7 (1.0,2.8)	1.0 (0.70,1.5)
*Missing*	*1.2* (*0.78,1.8*)			

Father's education		.022		
Secondary level	1.0		1.0	1.0
Primary level only	1.4 (1.0,1.8)		1.5 (0.94,2.2)	1.3 (0.93,1.9)
*Missing*	*1.4* (*0.94,2.1*)			

Housing quality		.001 (<.001)^b^		
Best	1.0		1.0	1.0
Intermediate	1.3 (1.0,1.7)		1.2 (0.78,1.9)	1.4 (0.98,2.0)
Worst	1.6 (1.3,2.2)		2.1 (1.4,3.1)	1.4 (0.96,2.0)
*Missing*	*1.4* (*0.94,2.1*)			

Care of house and child		.012 (.003)^b^		
Best	1.0		1.0	1.0
Intermediate	1.3 (0.95, 1.7)		1.5 (0.94,2.4)	1.1 (0.77,1.7)
Worst	1.5 (1.2,2.0)		1.8 (1.2,2.7)	1.3 (0.95,1.9)
*Missing*	*1.2* (*0.83,1.7*)			

**Most recent adult socio-economic conditions**				
Most recent household social class				
Non-manual	1.0	<.001	1.0	1.0
Manual	1.6 (1.3,2.1)		1.6 (1.1,2.3)	1.7 (1.2,2.3)
*Missing*	*2.5* (*1.8,3.4*)			

Most recent housing tenure				
Owner occupier	1.0	<.001	1.0	1.0
Other	2.8 (2.2,3.5)		3.1 (2.1,4.4)	2.6 (1.9,3.5)
*Missing*	*2.2* (*1.6,3.0*)			

Most recent household income				
Top two thirds	1.0	<.001	1.0	1.0
Bottom third	1.8 (1.4,2.2)		1.8 (1.3,2.6)	1.7 (1.2,2.4)
*Missing*	*1.7* (*1.3,2.4*)			

**Cigarette smoking**				
Never smoker	1.0	<.001	1.0	1.0
Former smoker	1.0 (0.76,1.5)		1.2 (0.68,2.0)	0.93 (0.62,1.4)
Current smoker	2.4 (1.8,3.1)		3.2 (2.1,4,8)	1.9 (1.3,2.7)
*Missing*	*2.3* (*1.5,3.5*)			

*p*-value = .026 for interaction between father's social class and sex. *p*-value > .05 for all other sex interactions.

a Test for heterogeneity.

b Test for trend calculated excluding missing category.

**Table 3:** Hazard ratios for mortality (26–60 years) for father's social class and care of house and child obtained from a Cox's proportional hazards model and based on 4461 study members and 332 deaths.

	Hazard ratio (95% CI) unadjusted	*p*-value^a^	Hazard ratio (95% CI) adjusted	*p*-value^a^
**All cause mortality**				
Father's social class & gender		<.001		.009
Non-manual, women	1.0		1.0	
Manual, women	2.2 (1.5,3.4)		1.9 (1.2,2.8)	
Non-manual, men	2.0 (1.3,3.2)		2.0 (1.3,3.0)	
Manual, men	2.5 (1.7,3.8)		2.0 (1.3,3.0)	
*Missing*	*2.4* (*1.5,4.1*)		*1.9* (*1.1,3.1*)	

Adult household social class		<.001		.07
Non-manual	1.0		1.0	
Manual	1.7 (1.3,2.1)		1.3 (0.98,1.6)	
*Missing*	*2.6* (*1.9,3.5*)		*2.8* (*1.5,5.1*)	

Housing tenure at 26		<.001		<.001
Owner occupier	1.0		1.0	
Other	2.8 (2.2,3.6)		2.4 (1.9,3.1)	
*Missing*	*2.2* (*1.6,3.0*)		*0.94* (*0.50,1.8*)	

**CVD mortality (*n*** **=** **81)**				
Father's social class & gender		.02		.09
Non-manual, women	1.0		1.0	
Manual, women	3.7 (1.3,10.8)		3.0 (1.0,8.6)	
Non-manual, men	3.9 (1.3,11.6)		3.7 (1.2,11.0)	
Manual, men	5.0 (1.8,14.2)		3.7 (1.3,10.5)	
*Missing*	*4.3* (*1.3,14.6*)		*3.0* (*0.86,10.3*)	

Adult household social class		.006		.17
Non-manual	1.0		1.0	
Manual	2.0 (1.2,3.3)		1.5 (0.85,2.5)	
*Missing*	*3.9* (*2.1,7.2*)		*2.4* (*0.72,8.2*)	

Housing tenure at 26		<.001		<.001
Owner occupier	1.0		1.0	
Other	3.2 (2.0,5.2)		2.6 (1.6,4.4)	
*Missing*	*3.5* (*2.0,6.3*)		*1.7* (*0.52,5.8*)	

**Cancer mortality (*n*** **=** **136)**				
Father's social class & gender		.004		.03
Non-manual, women	1.0		1.0	
Manual, women	2.4 (1.3,4.4)		2.1 (1.1,4.0)	
Non-manual, men	1.1 (0.53,2.4)		1.1 (0.52,2.3)	
Manual, men	2.3 (1.2,4.2)		2.0 (1.1,3.7)	
*Missing*	*2.8* (*1.3,5.9*)		*2.4* (*1.2,5.2*)	

Adult household social class		.029		.73
Non-manual	1.0		1.0	
Manual	1.5 (1.0,2.1)		1.1 (0.73,1.6)	
*Missing*	*1.5* (*0.88,2.7*)		*1.2* (*0.40,3.6*)	

Housing tenure at 26		<.001		<.001
Owner occupier	1.0		1.0	
Other	2.3 (1.6,3.4)		2.0 (1.4,3.0)	
*Missing*	*1.5* (*0.90,2.6*)		*1.3* (*0.44,3.6*)	

a Test for heterogeneity.

**Table 4:** Hazard ratios for mortality (26–60 years) for indicators of childhood cognitive ability at 8,11 and 15 years and educational attainment by 26 years obtained from Cox's proportional hazards models and based on 4461 men and women and 332 deaths.

	Total sample		Women	Men
	Hazard ratio (95% CI) sex adjusted	*p*-value^a^	Hazard ratio (95% CI) unadjusted	Hazard ratio (95% CI) unadjusted
**Cognitive ability age 8**		.012 (.001)^b^		
Lowest quarter	1.0		1.0	1.0
2	0.88 (0.65,1.2)		1.1 (0.71,1.8)	0.74 (0.50,1.1)
3	0.66 (0.48,0.91)		0.92 (0.56,1.5)	0.51 (0.33,0.79)
Highest quarter	0.62 (0.45,0.88)		0.66 (0.38,1.1)	0.61 (0.40,0.93)
*Missing* (*n* = *513*)	*0.88* (*0.61,1.3*)			

**Cognitive ability age 11**		.032 (.006)^b^		
Lowest quarter	1.0		1.0	1.0
2	0.75 (0.54,1.0)		0.86 (0.53,1.4)	0.67 (0.44,1.0)
3	0.75 (0.54,1.0)		0.78 (0.48,1.3)	0.73 (0.48,1.1)
Highest quarter	0.61 (0.44,0.86)		0.54 (0.31,0.93)	0.67 (0.43,1.0)
*Missing* (*n* = *691*)	*0.95* (*0.68,1.3*)			

**Cognitive ability age 15**		.004 (<.001)^b^		
Lowest quarter	1.0		1.0	1.0
2	0.75 (0.55,1.0)		0.77 (0.48,1.2)	0.73 (0.49,1.1)
3	0.62 (0.45,0.86)		0.63 (0.38,1.0)	0.61 (0.40,0.95)
Highest quarter	0.57 (0.40,0.80)		0.49 (0.28,0.85)	0.63 (0.41,0.98)
*Missing* (*n* = *691*)	*0.95* (*0.68,1.3*)			

**Childhood cognitive ability**		.004 (<.001)^b^		
Lowest quarter	1.0		1.0	1.0
2	0.71 (0.53,0.95)		0.75 (0.48,1.2)	0.68 (0.46,1.0)
3	0.59 (0.43,0.81)		0.67 (0.42,1.1)	0.54 (0.36,0.83)
Highest quarter	0.55 (0.40,0.76)		0.49 (0.29,0.82)	0.59 (0.39,0.89)
*Missing* (*333*)	*0.87* (*0.58,1.3*)			

**Educational qualifications by 26** **y**		.004 (.001)^b^		
None	1.0		1.0	1.0
Ordinary level	0.83 (0.62,1.1)		0.83 (0.55,1.3)	0.82 (0.53,1.3)
Advanced	0.63 (0.48,0.82)		0.48 (0.29,0.81)	0.70 (0.50,0.98)
*Missing* (*n* = *309*)	*1.2* (*0.82,1.8*)			

a Test for heterogeneity.

b Test for trend calculated excluding missing category.

**Table 5:** Hazard ratios for mortality (26–60 years) for childhood and early adulthood socio-economic conditions and for childhood cognitive ability obtained from a Cox's proportional hazards model and based on 4461 study members and 332 deaths.

	Hazard ratio (95% CI) Model a: unadjusted	*p*-value^a^	Hazard ratio (95% CI) Model b: adjusted	*p*-value^a^	Hazard ratio (95% CI) Model c: adjusted	*p*-value^a^
**Father's social class and gender**		<.001		.002		.01
Non-manual, women	1.0		1.0		1.0	
Manual, women	2.2 (1.5,3.4)		2.0 (1.3,3.1)		1.8 (1.2,2.8)	
Non-manual, men	2.0 (1.3,3.2)		2.0 (1.3,3.2)		2.0 (1.3,3.0)	
Manual, men	2.5 (1.7,3.8)		2.3 (1.5,3.4)		2.0 (1.3,3.0)	
*Missing*	*2.4* (*1.5,4.1*)		*2.1* (*1.2,3.6*)		*1.9* (*1.1,3.2*)	

**Childhood cognitive ability (standardised score)**				.02		.78
Lowest quarter			1.0		1.0	
2			0.74 (0.55,0.99)		0.88 (0.65,1.2)	
3			0.64 (0.47,0.89)		0.86 (0.62,1.2)	
Highest quarter			0.64 (0.46,0.91)		0.93 (0.64,1.3)	
*Missing*			*0.92* (*0.59,1.4*)		*0.91* (*0.58,1.4*)	

**Adult social class**						.13
Non-manual					1.0	
Manual					1.2 (0.94,1.6)	
*Missing*					*2.7* (*1.4,5.1*)	

**Adult housing tenure**						<.001
Owner occupier					1.0	
Other					2.4 (1.8,3.1)	
*Missing*					0.94 (0.49,1.8)	

Model a: father's social class and gender only. Model b: father's social class and gender, and childhood cognitive ability. Model c: father's social class and gender, childhood cognitive ability, adult social class and adult housing tenure.

a Test for heterogeneity.

**Table 6a:** Hazard ratios for mortality (26–60 years) for childhood and early adulthood socio-economic conditions and for childhood cognitive ability obtained from a Cox's proportional hazards model and based on 2325 men and 195 deaths.

	Hazard ratio (95% CI) Model a: unadjusted	*p*-value^a^	Hazard ratio (95% CI) Model b: adjusted	*p*-value^a^	Hazard ratio (95% CI) Model c: adjusted	*p*-value^a^	Hazard ratio (95% CI) Model d: adjusted	*p*-value^a^
Father's social class		.17		.63		.92		.86
Non-manual	1.0		1.0		1.0		1.0	
Manual	1.2 (0.91,1.7)		1.1 (0.78,1.5)		0.98 (0.70,1.4)		0.97 (0.69,1.4)	
*Missing*	*1.3* (*0.77,2.2*)		*1.1* (*0.60,1.9*)		*0.93* (*0.53,1.7*)		*0.90* (*0.51,1.6*)	

Childhood cognitive ability (standardised score)				.03		.56		.53
Lowest quarter			1.0		1.0		1.0	
2			0.68 (0.46,1.0)		0.80 (0.54,1.2)		0.79 (0.53,1.2)	
3			0.56 (0.36,0.86)		0.75(0.48,1.2)		0.77 (0.49,1.2)	
Highest quarter			0.61 (0.40,0.95)		0.89 (0.55,1.4)		0.92 (0.57,1.5)	
*Missing*			*0.94* (*0.54,1.61*)		*0.92* (*0.53,1.6*)		*1.0* (*0.57,1.8*)	

Adult social class						.14		.22
Non-manual					1.0		1.0	
Manual					1.3 (0.92,1.9)		1.3 (0.87,1.8)	
*Missing*					*2.4* (*1.1,5.4*)		*2.6* (*1.2,5.8*)	

Adult housing tenure						<.001		<.001
Owner occupier					1.0		1.0	
Other					2.2 (1.6,3.1)		2.0 (1.4,2.8)	
*Missing*					*1.1* (*0.50,2.4*)		*0.98* (*0.41,2.3*)	

Smoking behaviour								.02
Never smoker							1.0	
Former smoker							1.0 (0.69,1.6)	
Current smoker							1.6 (1.1,2.3)	
*Missing*							*1.1* (*0.52,2.1*)	

Model a: father's social class only. Model b: father's social class and childhood cognitive ability. Model c: father's social class, childhood cognitive ability, adult social class and adult housing tenure. Model d: father's social class, childhood cognitive ability, adult social class, adult housing tenure and smoking behaviour.

a Test for heterogeneity.

**Table 6b:** Hazard ratios for mortality (26–60 years) for childhood and early adulthood socio-economic conditions and for childhood cognitive ability obtained from a Cox's proportional hazards model and based on 2136 women and 137 deaths.

	Hazard ratio (95% CI) Model a: unadjusted	*p*-value^a^	Hazard ratio (95% CI) Model b: adjusted	*p*-value^a^	Hazard ratio (95% CI) Model c: adjusted	*p*-value^a^	Hazard ratio (95% CI) Model d: adjusted	*p*-value^a^
Father's social class		<.001		.002		.005		.009
Non-manual	1.0		1.0		1.0		1.0	
Manual	2.2 (1.5,3.4)		2.0 (1.3,3.1)		1.9 (1.2,3.0)		1.8 (1.2,2.9)	
*Missing*	*2.2* (*1.1,4.3*)		*2.0* (*0.95,4.1*)		*1.9* (*0.90,3.9*)		*1.7* (*0.83,3.7*)	

Childhood cognitive ability (standardised score)				.53		>.99		.96
Lowest quarter			1.0		1.0		1.0	
2			0.82 (0.53,1.3)		1.0 (0.63,1.6)		1.0 (0.66,1.7)	
3			0.78 (0.48,1.3)		1.0 (0.63,1.7)		1.1 (0.69,1.9)	
Highest quarter			0.68 (0.39,1.2)		1.0 (0.56,1.8)		1.1 (0.61,2.0)	
*Missing*			*0.88* (*0.41,1.9*)		*0.89* (*0.40,1.9*)		*0.92* (*0.42,2.0*)	

Adult social class						.54		.97
Non-manual					1.0		1.0	
Manual					1.1 (0.77,1.7)		1.0 (0.68,1.5)	
*Missing*					*3.6* (*1.2,10.3*)		*3.8* (*1.3,11.6*)	

Adult housing tenure						<.001		<.001
Owner occupier					1.0		1.0	
Other					2.6 (1.8,3.9)		2.2 (1.5,3.3)	
*Missing*					*0.66* (*0.22,2.0*)		*0.43* (*0.11,1.5*)	

Smoking behaviour								<.001
Never smoker							1.0	
Former smoker							1.2 (0.69,2.0)	
Current smoker							2.5 (1.7,3.9)	
*Missing*							*2.2* (*0.85,5.8*)	

Model a: father's social class only. Model b: father's social class and childhood cognitive ability. Model c: father's social class, childhood cognitive ability, adult social class and adult housing tenure. Model d: father's social class, childhood cognitive ability, adult social class, adult housing tenure and smoking behaviour.

a Test for heterogeneity.

## References

[bib1] Batty G.D., Der G., MacIntyre S., Deary I.J. (2006). Does IQ explain socioeconomic inequalities in health? Evidence from a population based cohort study in the west of Scotland. British Medical Journal.

[bib2] Batty G.D., Mortensen E.L., Nybo Andersen A.M., Osler M. (2005). Childhood intelligence in relation to adult coronary heart disease and stroke risk: evidence from a Danish birth cohort study. Paediatric and Perinatal Epidemiology.

[bib3] Bengtsson T., Brostrom G. (2006). Old-age mortality in a life-course perspective. http://paa2006.princeton.edu/abstractViewer.aspx%3FsubmissionId%3D60997.

[bib4] Blane D. (2003). Commentary: explanations of the difference in mortality risk between different educational groups. International Journal of Epidemiology.

[bib6] Chen Y., Horne S.L., Dosman J.A. (1991). Increased susceptibility to lung dysfunction in female smokers. American Review of Respiratory Disease.

[bib7] Davey Smith G., Hart C., Blane D., Gillis C., Hawthorne V. (1997). Lifetime socioeconomic position and mortality: prospective observational study. British Medical Journal.

[bib8] Davey Smith G., Hart C., Blane D., Hole D. (1998). Adverse socioeconomic conditions in childhood and cause specific adult mortality: prospective observational study. British Medical Journal.

[bib9] Davey Smith G., McCarron P., Okasha M., McEwen J. (2001). Social circumstances in childhood and cardiovascular disease mortality: prospective observational study of Glasgow University students. Journal of Epidemiology and Community Health.

[bib10] Deary I.J., Batty G.D. (2006). Commentary: pre-morbid IQ and later health–the rapidly evolving field of cognitive epidemiology. International Journal of Epidemiology.

[bib11] Deary I.J., Der G. (2005). Reaction time explains IQ's association with death. Psychological Science.

[bib12] Doll R., Peto R., Boreham J., Sutherland I. (2004). Mortality in relation to smoking: 50 years' observations on male British doctors. British Medical Journal.

[bib13] Douglas J.W.B. (1964). The home and the school.

[bib14] Elo I.T., Preston S.H. (1992). Effects of early-life conditions on adult mortality: a review. Population Index.

[bib15] Galobardes B., Lynch J.W., Davey Smith G. (2004). Childhood socioeconomic circumstances and cause-specific mortality in adulthood: systematic review and interpretation. Epidemiologic Reviews.

[bib16] Gliksman M.D., Kawachi I., Hunter D., Colditz G.A., Manson J.E., Stampfer M.J. (1995). Childhood socioeconomic status and risk of cardiovascular disease in middle aged US women: a prospective study. Journal of Epidemiology and Community Health.

[bib17] Halsey H.H., Webb J. (2000). Twentieth century British social trends.

[bib18] Hart C.L., Taylor M.D., Davey Smith G., Whalley L.J., Starr J.M., Hole D.J. (2005). Childhood IQ and all-cause mortality before and after age 65: prospective observational study linking the Scottish mental survey 1932 and the midspan studies. British Journal of Health Psychology.

[bib19] Hemmingsson T., Lundberg I. (2005). Can large relative mortality differences between socio-economic groups among Swedish men be explained by risk indicator-associated social mobility?. European Journal of Public Health.

[bib20] Hemmingsson T., Melin B., Allebeck P., Lundberg I. (2006). The association between cognitive ability measured at ages 18–20 and mortality during 30 years of follow-up–a prospective observational study among Swedish males born 1949–51. International Journal of Epidemiology.

[bib21] Kuh D., Hardy R., Langenberg C., Richards M., Wadsworth M.E. (2002). Mortality in adults aged 26–54 years related to socioeconomic conditions in childhood and adulthood: post war birth cohort study. British Medical Journal.

[bib22] Kuh D., Richards M., Hardy R., Butterworth S., Wadsworth M.E. (2004). Childhood cognitive ability and deaths up until middle age: a post-war birth cohort study. International Journal of Epidemiology.

[bib23] Lawlor D.A., Sterne J.A., Tynelius P., Davey Smith G., Rasmussen F. (2006). Association of childhood socioeconomic position with cause-specific mortality in a prospective record linkage study of 1,839,384 individuals. American Journal of Epidemiology.

[bib24] Martin L.T., Kubzansky L.D. (2005). Childhood cognitive performance and risk of mortality: a prospective cohort study of gifted individuals. American Journal of Epidemiology.

[bib25] Mirowsky J., Ross C.E. (2003). Education, social status and health.

[bib26] Neisser U., Boodoo G., Bouchard T., Boykin A., Brody N., Ceci S. (1996). Intelligence: knowns and unknown. American Psychologist.

[bib27] O'Toole B.I., Stankov L. (1992). Ultimate validity of psychological tests. Personality and Individual Differences.

[bib28] Osler M., Andersen A.M., Due P., Lund R., Damsgaard M.T., Holstein B.E. (2003). Socioeconomic position in early life, birth weight, childhood cognitive function, and adult mortality. A longitudinal study of Danish men born in 1953. Journal of Epidemiology and Community Health.

[bib29] Pearlin L.I., Aneshensel C., Phelan J. (1999). The stress process revisited. Handbook of the sociology of mental health.

[bib30] Peto R., Lopez A.D., Boreham J., Thun M., Heath C. (1992). Mortality from tobacco in developed countries: indirect estimation from national vital statistics. Lancet.

[bib31] Phelan J.C., Link B.G. (2005). Controlling disease and creating disparities: a fundamental cause perspective. Journal of Gerontology B Psychological Sciences and Social Sciences.

[bib32] Pigeon D.A., Douglas J.W.B. (1964). Tests used in the 1954 and 1957 surveys. The home and the school.

[bib33] Pigeon D.A., Douglas J.W.B., Ross J.M., Simpson H.R. (1968). Details of the fifteen years tests. All our future.

[bib34] Pollitt R.A., Rose K.M., Kaufman J.S. (2005). Evaluating the evidence for models of life course socioeconomic factors and cardiovascular outcomes: a systematic review. BMC. Public Health.

[bib35] Pope M., Ashley M.J., Ferrence R. (1999). The carcinogenic and toxic effects of tobacco smoke: are women particularly susceptible?. Journal of Gender-Specific Medicine.

[bib36] Power C., Graham H., Due P., Hallqvist J., Joung I., Kuh D., Lynch J. (2005). The contribution of childhood and adult socioeconomic position to adult obesity and smoking behaviour: an international comparison. International Journal of Epidemiology.

[bib37] Power C., Kuh D., Siegrist J., Marmot M. (2006). Life course development of unequal health. Social inequalities in health. New evidence and policy implications.

[bib38] Richards M., Sacker A. (2003). Lifetime antecedents of cognitive reserve. Journal of Clinical and Experimental Neuropsychology.

[bib39] Richards M., Shipley B., Fuhrer R., Wadsworth M.E. (2004). Cognitive ability in childhood and cognitive decline in mid-life: longitudinal birth cohort study. British Medical Journal.

[bib40] Shipley B.A., Der G., Taylor M.D., Deary I.J. (2006). Cognition and all-cause mortality across the entire adult age range: health and lifestyle survey. Psychosomatic Medicine.

[bib41] Stabile L.P., Siegfried J.M. (2003). Sex and gender differences in lung cancer. Journal of Gender-Specific Medicine.

[bib42] Strachan D.P., Perry I.J., Kuh D., Ben-Shlomo Y. (1997). Time trends. A life course approach to chronic disease epidemiology.

[bib43] Taylor M.D., Hart C.L., Davey Smith G., Starr J.M., Hole D.J., Whalley L.J. (2003). Childhood mental ability and smoking cessation in adulthood: prospective observational study linking the Scottish mental survey 1932 and the midspan studies. Journal of Epidemiology and Community Health.

[bib44] Townsend J., Roderick P., Cooper J. (1994). Cigarette smoking by socioeconomic group, sex, and age: effects of price, income, and health publicity. British Medical Journal.

[bib45] Vollset S.E., Tverdal A., Gjessing H.K. (2006). Smoking and deaths between 40 and 70 years of age in women and men. Annals of Internal Medicine.

[bib46] Wadsworth M.E., Butterworth S.L., Hardy R.J., Kuh D.J., Richards M., Langenberg C. (2003). The life course prospective design: an example of benefits and problems associated with study longevity. Social Science & Medicine.

[bib47] Whalley L.J., Deary I.J. (2001). Longitudinal cohort study of childhood IQ and survival up to age 76. British Medical Journal.

